# Gut Microbiota in Depression: A Focus on Ketamine

**DOI:** 10.3389/fnbeh.2021.693362

**Published:** 2021-06-23

**Authors:** Alina Wilkowska, Łukasz Piotr Szałach, Wiesław Jerzy Cubała

**Affiliations:** Medical University of Gdansk, Gdańsk, Poland

**Keywords:** ketamine, S-ketamine, R-ketamine, gut microbiota, gut-brain axis, treatment resistance, inflammation, HPA axis

## Abstract

According to the WHO, major depressive disorder is the leading cause of disability worldwide, and it is a major contributor to the overall global burden of disease. The pathophysiology of this common and chronic disease is still not completely understood. The gut microbiome is an increasingly recognized environmental factor that can have a role in depression, acting through the gut–microbiota–brain axis. The available treatment for depression is still insufficient since 30% of patients are treatment-resistant. There is an unquestionable need for novel strategies. Ketamine is an effective antidepressant in treatment-resistant patients. It is suggested that the antidepressant effect of ketamine may be partially mediated by the modification of gut microbiota. In this study, we presented a review of data on gut microbiota in depression with special attention to the effect of ketamine on the microbiome in animal models of depression. Earlier reports are preliminary and are still insufficient to draw firm conclusion, but further studies in this field might help to understand the role of the gut–brain axis in the treatment of depression and might be the ground for developing new effective treatment strategies.

## Introduction

Major depressive disorder (MDD) is a severe, recurrent disease affecting more than 264 million people of the world population (Smith, 2014; World Health Organization., 2017a). Recent data show that ~800,000 people with depression commit suicide every year (World Health Organization., 2017b). Depression correlates with disturbances in hypothalamic–pituitary–adrenal (HPA) axis (Barden, 2004) and causes increased inflammatory response (Dantzer et al., 2008; Miller et al., 2009). Increasing evidence suggests that microbiota plays a significant role in development (O'Mahony et al., 2009; Zheng et al., 2016) and possibly in the treatment of depression (Liu et al., 2019). The available treatment strategies for MDD are still insufficient. About 30% of patients are treatment-resistant (Rush et al., 2006). One of the new treatments involves *N*-methyl-d-aspartate receptor (NMDAR) antagonist, namely, ketamine. The mechanism of the antidepressant effect of ketamine is still not fully understood. Studies have indicated that it involves the inhibition of presynaptic and postsynaptic NMDARs in gamma-aminobutyric acidergic (GABAergic) interneurons. The mechanism also activates postsynaptic α-amino-3-hydroxy-5-methyl-4 isoxazole propionic acid receptors (AMPARs) and the brain-derived neurotrophic factor-tyrosine receptor kinase B (BDNF-TrkB) signaling pathway. Ketamine and its enantiomers have a rapid antidepressant and antisuicidal effect as an add-on treatment in unipolar and bipolar treatment-resistant depression (TRD) (Park et al., 2019; Wilkowska et al., 2020). There is an increasing number of evidence that the gut microbiota may play a crucial role in the antidepressant effects of ketamine (Wang et al., 2020).

In this review, first, we introduced the concept of gut–brain axis (GBA) and the studies on which it is based. We presented studies on germ-free (GF) and specific pathogen-free (SPF) mice, using vagotomy, probiotics, and antibiotics. We also discussed the role of the intestinal barrier and blood–brain barrier (BBB) in the GBA. Then, we demonstrated animal and human studies on the effect of depression on the gut microbiome and the functioning of gut–brain barrier. The second part of this review concentrates on ketamine. We started with the antibacterial and anti-inflammatory effects of ketamine and then presented studies on microbiota changes as an effect of ketamine use in animal models of depression. Recently, some reviews that are more informative have been published in this field, but none of them mentions ketamine and its potential role of regulating GBA in major depression (Barandouzi et al., 2020; Yang et al., 2020; Simpson et al., 2021).

## Gut–Brain Axis

Human gut microbiota consists of trillions of bacteria that are critical for nutrition and also significantly affect the central nervous system (CNS) (Hooper et al., 2001; Macpherson and Harris, 2004). Studies prove that there is bidirectional communication between brain and gut, and this process is captivated by the term GBA (Cryan et al., 2018). This communication system uses three main pathways, namely, neural—enteric nerves and vagus nerve, inflammatory—cytokines and immune cells, and humoral—HPA axis. Clinical studies suggest that the role of GBA is in the pathophysiology of irritable bowel syndrome (IBS) and of mental disorders such as autism, schizophrenia, Parkinson's disease, Alzheimer's disease, anxiety disorders, and MDD (Cryan et al., 2018).

In order to study pathological microbiota composition, it is necessary to know what defines a healthy gut microbiome. The normal human gut microbiota comprises two major phyla, namely, Bacteroidetes and Firmicutes. The next two most prominent phyla are Actinobacteria and Verrucomicrobia. The composition of microbiota stabilizes by the age of three, and at this point, it resembles the adult microbiome, although it is not constant and can undergo some changes due to the effect of various factors. A healthy colon also hosts primary pathogens such as *Campylobacter jejuni, Salmonella enterica, Vibrio cholerae*, and *Escherichia coli*, but in very low abundance (Jandhyala et al., 2015).

The current knowledge on GBA comes mainly from animal studies, specifically the ones involving GF mice. This model allows for studying mice in the absence of microbes and for comparing the observed processes in colonized mice, thereby unrevealing the effect of gut microbiota on the brain. There are two models of colonization used in such studies. The first model is SPF environment in which mice are raised with normal functional microbiota, but they are free of pathogens that could disturb their health and research outcomes. The second mice model involves gnotobiotic mice that are simply GF mice inoculated intentionally with known non-pathogenic microorganisms. Sudo et al. compared GF, SPF, and gnotobiotic mice to investigate the effect of microbes on stress response and neuroplasticity. The results have shown increased stress response with higher levels of adrenocorticotropic hormone (ACTH) and cortisol in GF compared with SPF mice. Moreover, the recolonization of the gut with *Bifidobacterium infantis* normalized the HPA axis, but only in young mice, suggesting that the brain is susceptible to microbiota effect at a specific time during development. This observation suggests that commensal microbiota is an environmental determinant that programs the HPA stress response (Sudo et al., 2004). The authors also found that GF male mice had the reduced BDNF and 2A subtype of NMDAR expression in the brain cortex and hippocampus compared with SPF mice (Clarke et al., 2013). Another study has demonstrated that GF male mice have significantly elevated hippocampal serotonin levels compared with conventionally colonized mice, and this change is resistant to the restoration of normal gut flora. It has also been found that mRNA of hippocampal BDNF in GF mice is reduced compared with their conventionally colonized counterparts, but this effect is present only in male animals. Interestingly, this effect is sex-specific, suggesting that the regulation of gut–microbiota–brain axis may be dependent on gender (Clarke et al., 2013). Diaz et al. have observed increased motor activity and reduced anxiety-like behavior in GF mice. This effect was accompanied by increased serotonin, noradrenaline, and dopamine turnover in the striatum of GF mice compared with SPF mice. The authors also found decreased expression of neuroplasticity genes, such as BDNF and nerve growth factor-inducible clone A (NGFI-A), in the brain of GF mice compared with SPF mice. Another noteworthy observation was the decreased expression of synaptic-related proteins (e.g., synaptophysin and PSD-95) in the striatum of SPF and conventionally raised mice compared with GF mice, which suggests that microbiota modulates the sensitive period of synaptogenesis. All these results suggest that gut microbiota is involved in the programming of neuronal circuits and therefore affects behavior (Diaz Heijtz et al., 2011). A study has shown an increased adult hippocampal neurogenesis in GF mice compared with conventionally colonized mice. The recolonization of GF mice did not prevent changes in adult hippocampal neurogenesis, again suggesting that there is a critical window in early life during which gut microbiota influences this process (Ogbonnaya et al., 2015). Another study supporting the role of bacteria in the bidirectional communication of the GBA used *Lactobacillus rhamnosus* (JB-1) as an active agent and compared the expression of GABA receptors in various regions of the brain between treatment group and conventionally fed mice. The authors observed mRNA alterations in region-dependent GABA receptors. Moreover, they also found reduced stress-induced corticosterone and anxiety- and depression-related behaviors in the treatment group. Neurochemical and behavioral effects were not found in vagotomized mice, and this identifies the vagus nerve as one of the main communication pathways between gut microbiota and the brain (Bravo et al., 2011).

Beside humoral and neural pathways, GBA also uses the immune system for this bidirectional communication (Hooper et al., 2012). Microbiota has a critical role in the development of organized lymphoid structures and in the function of immune system cells. Gut microbes modulate the maturation and function of immune cells in the CNS, such as microglia, and also in peripheral immune cells (Fung et al., 2017).

An alternative way of investigating microbiota and its role in communication with the CNS is to use antibiotics in order to change microbiota composition and observe its effect on brain function and behavior in animals. A study found that the administration of a mixture of oral non-absorbable antimicrobials to SPF mice caused a significant increase in Firmicutes and Actinobacteria and a decrease in Gammaproteobacteria and Bacteroidetes. This change was associated with increased exploratory behavior and increased hippocampal BDNF levels. These effects were reversible after the withdrawal of antibiotics. Oral antibiotic administration did not cause any changes in behavior in GF mice, which suggests that the presence of microbiota is necessary for this effect. The authors performed additional tests and found that the effect of antibiotics was independent of the autonomic nervous system, inflammation, and gastrointestinal neurotransmitters. They suggested that there must be another way of communicating between the gut and the brain, and this way may involve the production of neuroactive substances by bacteria (Bercik et al., 2011).

Evidence from clinical and animal studies shows that enteric microbiota has a significant impact on GBA, interacting directly with enterocytes and enteric neurons (Carabotti et al., 2015). Certain metabolites from gut microbes alter neurotransmitter production in the cells of the colon (Yano et al., 2015; Kiraly et al., 2016). On the other hand, the brain acts on gastrointestinal and immune functions and affects gut microbiota composition in this way (Ha et al., 2014; Carabotti et al., 2015). Furthermore, gut microbes may produce molecules that can act as local neurotransmitters, such as GABA, serotonin, melatonin, histamine, and acetylcholine (Iyer et al., 2004). They can also transform catecholamine substrates to a biologically active form (Asano et al., 2012). It has been reported that binding sites for enteric neurotransmitters are present in bacteria and can influence the function of components of the microbiota (Hughes and Sperandio, 2008).

The interaction of microbiota and GBA might also occur through the release of biologically active peptides from enteroendocrine cells. The digestive tract is a source of regulatory peptides that act locally on the epithelial cells and the enteric nervous system and also have distant targets in the brain (Uribe et al., 1994). For example, galanin stimulates the release of cortisol-releasing factor (CRF) and ACTH, thereby enhancing cortisol secretion. Galanin also seems to stimulate cortisol secretion directly from adrenocortical cells and norepinephrine release from the adrenal medulla in rats (Tortorella et al., 2007). A human study suggests that ghrelin, another psychoactive peptide, has a marked ACTH/CRF effect, and it is probably involved in the modulation of the HPA response to stress (Giordano et al., 2006). Recent studies on GF and conventionalized mice have proved that one of the major human symbionts, *Bacteroides thetaiotaomicron*, promotes neurogenesis in the enteric nervous system and regulates enteroendocrine networks through its major fermentation products, acetate, propionate, and succinate (Aktar et al., 2020; Modasia et al., 2020).

## Microbiota and Intestinal Barrier

The intestinal epithelium acts as a barrier between the host and the commensal bacteria, enabling their symbiotic relationship. Enterocytes form the physical barrier by linking together with various cell junctions such as desmosomes, adherens junctions, and tight junctions (Hiippala et al., 2018). Tight junctions, such as claudins, occluding, and intercellular junctions, control the paracellular permeability and moderate the transepithelial transport (Ulluwishewa et al., 2015). Microbiota dysbiosis can impair the epithelial barrier leading to the so-called “leaky gut,” allowing the intestinal content to be in contact with the host periphery, potentially inducing inflammatory response (Walker and Lawley, 2013). There is evidence that the probiotic *Bacteroides fragilis* normalizes increased intestinal epithelial permeability in a mouse model of autism spectrum disorders (Hsiao et al., 2013). Studies suggest that probiotic bacteria enhance the intestinal barrier, causing changes in the tight junction protein expression and distribution. Commensal bacteria, together with intestinal inflammation and dietary components, are the main factors affecting epithelial permeability (Suzuki, 2013).

## Microbiota and Blood–Brain Barrier

Blood–brain barrier controls the passage and exchange of molecules and nutrients between the circulatory system and the brain cells. The development of the brain includes the formation of intact BBB, which promises optimal conditions for neuronal growth and cell specification (Engelhardt, 2003). BBB is formed by capillary endothelial cells sealed by tight junctions, astrocytes, and pericytes. Tight junction proteins consist of transmembrane proteins such as claudins, tricellulin, and occludin (Tscheik et al., 2013). There is evidence that lack of gut microbiota is associated with increased BBB permeability and altered expression of tight junction proteins (Braniste et al., 2014). It was also found that fecal transfer from mice with pathogen-free gut flora into GF mice or treatment of GF mice with bacteria that produce short-chain fatty acids (SCFAs) decreases the permeability of the BBB (Thabane et al., 2010). Although the study has not revealed the precise signaling mechanisms through which gut microbiota modulates BBB function, it sheds light on another aspect of microbiota affecting the brain.

Another proof for microbiota regulating GBA was obtained from a population-based study from Walkerton in Canada, according to which, as the consequence of a flood in the year 2000, drinking water was contaminated with *E. coli* and *C. jejuni*. This contamination caused gastrointestinal infections in more than 2,000 inhabitants. Thabane et al. followed over 400 children for 7 years and found that acute bacterial gastroenteritis was associated with more than 4-fold increase in the incidence of IBS among children exposed to acute gastroenteritis compared with unexposed controls (Thabane et al., 2010).

## Microbiome in Depression

There is a strong connection between chronic stress, microbiota changes, activation of the inflammatory system, and depression. It has been shown that the levels of pro-inflammatory cytokines, mainly the interleukins (IL), such as, IL-1, IL-6, IFN-γ, and TNF-α, are elevated in the serum of patients suffering from depression (Dowlati et al., 2010; Schmidt et al., 2014; Haapakoski et al., 2015). Elevated serum concentrations of other cytokines, such as IL-5, IL-7, IL-8, IL-10, IL-12, and IL-13, have also been reported (Schmidt et al., 2014).

A preclinical study revealed significant changes of the gut microbiome in rats subjected to maternal separation compared with control (O'Mahony et al., 2009). Another study has shown that microbiome composition in mice exposed to the long-term restraint stress was also significantly altered compared with nonstressed mice (Bangsgaard Bendtsen et al., 2012). Bailey et al. have shown that mice exposed to a social stressor called social disruption had lower relative abundance of genus *Bacteroides* and higher relative abundance of genus *Clostridium*. The microbial diversity of stressed mice was significantly reduced. Moreover, stressor-induced increase in levels of circulating IL-6 and monocyte chemoattractant protein-1 (MCP-1) correlated with stressor-induced changes in three members of the microbiota, such as *Dorea* spp., *Coprococcus* spp., and *Pseudobutyrivibrio* spp. (Bailey et al., 2011).

Inflammasome, a protein complex that generates and augments stress-induced immune response *via* activation of caspase-1 and increased IL-1beta and IL-18 secretion, is considered to play an important role in the development of depression. It has been proved that caspase-1 knockout mice, apart from significantly lower depressive-like behaviors and lower IL-1beta and IL-18 levels, presented several microbiota changes in comparison with mice with activated inflammasome (Wong et al., 2016). Zhang et al. tested the link between IL-6 and microbiota in a mouse model of depression. The authors used the anti-mouse IL-6 receptor antibody (MR16-1) and social defeat stress (SDS) model. Susceptible mice presented gut microbiota alterations. MR16-1 had an antidepressant effect, but it also improved decreased Firmicutes/Bacteroidetes ratio at the phylum level and significantly improved the increased levels of *Sutterella* and decreased levels of *Oscillospira* at the genus level in susceptible mice after SDS. These findings suggest that the blockade of IL-6 receptor in the periphery might have an antidepressant effect, achieved through normalizing the altered composition of gut microbiota in susceptible mice after SDS (Zhang et al., 2017).

Zheng et al. demonstrated that the absence of gut microbiota induces depression-like behavior in mice. The human part of the study confirmed that the composition of gut microbiota in patients with MDD was significantly altered compared with control. This effect was characterized by the alterations in Firmicutes, Actinobacteria, and Bacteroidetes at the phylum level. In order to determine whether gut microbiota have a role in developing depression, they colonized GF mice with microbiota from depressed patients. This process caused increased depression-like behaviors in mice (Zhang et al., 2017). Another similar study demonstrated that the oral transplantation of gut microbiota from depressed patients to microbiota-depleted rats induces depressive-like, anhedonic behavioral phenotype with a simultaneous increase in acute phase proteins and altered tryptophan metabolism. The authors suggested that the gut microbiota may play a role in the development of depression most probably through the immunomodulatory pathway and may provide a target in the treatment and prevention of this disorder (Kelly et al., 2016).

Another environmental factor that can modulate GBA in depression is diet; however, this subject is beyond the scope of this review (Aly and Engmann, 2020; Włodarczyk et al., 2021).

## Probiotics in Studies on Depression

The next set of data on the role of gut microbiota in depression comes from studies with probiotics. Probiotics are microorganisms that contribute to the host gut microbial flora when consumed and produce beneficial effects on health. In animal models of depression, chronic probiotic administration can reduce anxiety and depressive symptoms and correlates with the normalization of biological indicators of depression, such as corticosterone, noradrenaline, BDNF levels, and cytokines (Desbonnet et al., 2010; Bercik et al., 2011; Bravo et al., 2011). Some probiotics such as *L. helveticus* R0052 and *B. longum* R0175 seem to restore tight-junction integrity in the intestinal barrier and also affect hippocampal neurogenesis (Ait-Belgnaoui et al., 2014). One of the studies found that the supplementation of *Bifidobacterium* in mice causes resilience to chronic social defeat stress (CSDS), which is an animal model of depression (Yang et al., 2017a).

A recent systematic meta-analysis including 29 trials of probiotics in humans shows that probiotics have a small, but significant, effect on depression and anxiety, although the number of trials with clinical samples is still not sufficient. Most of the trials investigated *Lactobacilli* alone or in combination with species from other genera, most often *Bifidobacterium* (Liu et al., 2019).

## Gut Microbiota in MDD

The impact of altered microbiota on GBA in depression is the subject of scientific interest (Dinan and Cryan, 2019). The first clinical study investigating gut microbiota in depressed patients vs. controls found no significant differences in diversity between the two groups, but several correlations between depression and the fecal microbiota were observed (Naseribafrouei et al., 2014). Jiang et al. analyzed fecal samples from 46 patients with depression and 30 healthy controls. They found significant differences in microbiota composition of patients with MDD. Bacteroidetes, Proteobacteria, and Actinobacteria were increased, while Firmicutes was decreased, in MDD subjects compared with healthy controls. Most notably, the MDD groups had increased levels of Enterobacteriaceae and *Alistipes* but reduced levels of *Faecalibacterium*. A negative correlation was observed between *Faecalibacterium* and the severity of depressive symptoms (Jiang et al., 2015). Another recent study involving 40 patients with practitioner-reported depression and 70 healthy controls (i.e., sample extracted from the cohort of Flemish Gut Flora Project; *n* = 1,054) found that two bacterial genera, namely, *Coprococcus* and *Dialister*, were depleted in patients with depression diagnosed by their general practitioner (Valles-Colomer et al., 2019). A recent systematic meta-analysis included two already mentioned reports (Naseribafrouei et al., 2014; Jiang et al., 2015) and additional eight observational studies and found that, at the phylum level, the findings were inconsistent. At the family level, Veillonellaceae, Prevotellaceae, and Sutterellaceae were less abundant in patients with MDD than in non-depressed controls, and Actinomycetaceae was elevated in those with MDD than in controls. At the genus level, *Coprococcus, Faecalibacterium, Ruminococcus, Bifidobacterium*, and *Escherichia* were reduced in patients with MDD than in non-depressed controls, whereas *Paraprevotella* was increased in depressed patients (Sanada et al., 2020).

## Gut–Brain Barrier in MDD

**Figure 1:**
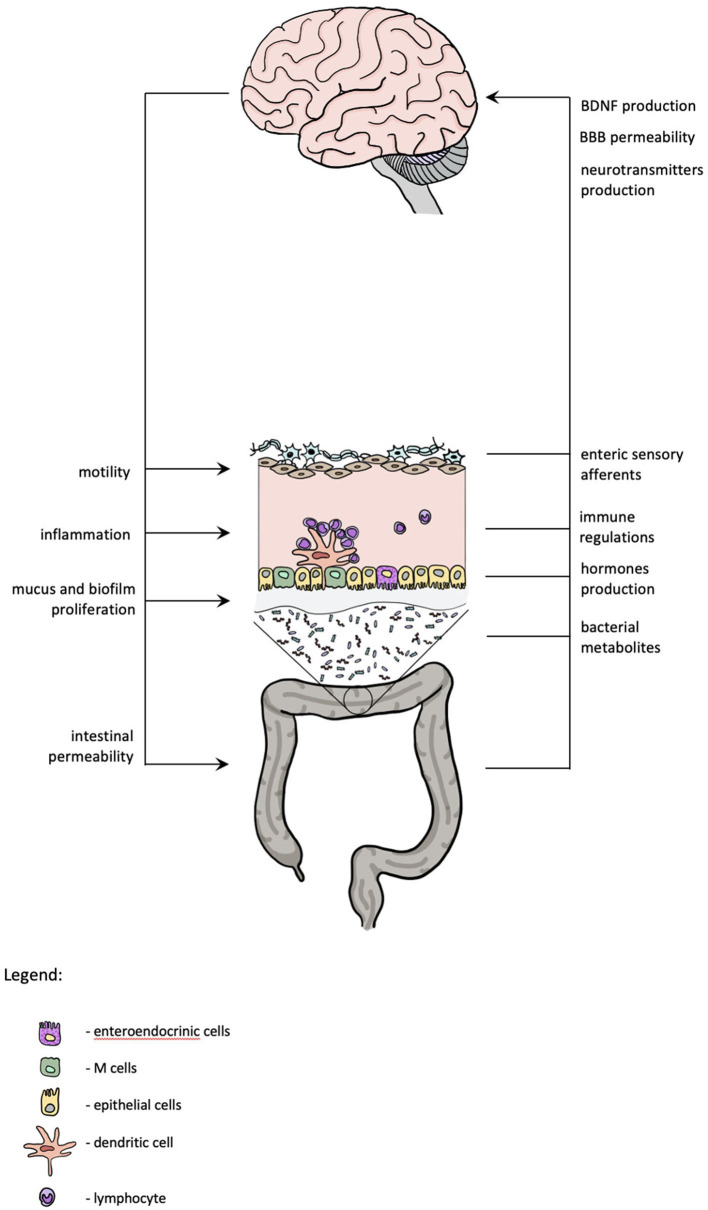
Gut-brain axis and depression. The scheme summarizes the mutual interactions between central nervous system and gastrointestinal system. Functional changes in both might contribute to depression.

Major depressive disorder is associated with an increased translocation of bacterial products from the gut. According to the “leaky gut hypothesis,” increased intestinal permeability in depressed patients may contribute to inflammatory response *via* bacterial translocation across the enterocytes. Maes et al. found that depressed patients have increased IgA and IgM response against lipopolysaccharide (LPS)—part of the wall of gram-negative commensal bacteria (Maes et al., 2012). LPS is recognized by the CD14-Toll-like receptors expressed by peripheral blood mononuclear cells (PBMCs) and also by neurons, microglia, and astrocytes. In depression, bacteria translocate from the epithelium to lamina propria and mesenteric lymph nodes (i.e., site of antigen presentation), which may then activate PBMCs and provoke immunoglobulin production (Maes et al., 2008). It also seems that increased bacterial translocation is related to the level of oxidative and nitrosative stress in depressed patients (Maes et al., 2013). A study on gut permeability in patients with recent suicide attempts in course of MDD found that permeability markers—zonulin and intestinal fatty acid-binding protein (I-FABP)—are altered in patients with recent suicide attempt compared with controls. These markers correlated with the IL-6 levels and I-FABP concentration correlated with the severity of depressive symptoms. The authors suggested that “leaky gut hypothesis” may elucidate the association between inflammation and suicidal behavior (Ohlsson et al., 2019). Increased release of IL-6, IL-1β, and TNFα can lead to alterations in central neurotransmission and cause symptoms called “sickness behavior” (D'Mello and Swain, 2017). Patients with inflammatory disorders, such as chronic liver disease, IBS, and rheumatoid arthritis, have high comorbidity of depression and sickness behavior, although the distinction between both is difficult due to overlap of symptoms such as fatigue, increased anxiety, loss of appetite, sleep disturbances, and loss of social interest (D'Mello and Swain, 2014). The elements of gut-brain axis which might have a role in depression are presented in Figure 1.

## Gut Microbiota and Antidepressants

An increasing number of evidence indicates that drugs affect microbiota composition. Among them, there are psychotropic drugs including antidepressants (Cussotto et al., 2019). According to *in vitro* studies, sertraline is a strong antimicrobial agent which inhibits the growth of *Staphylococcus aureus, E. coli*, and *Pseudomonas aeruginosa*, and it also augments the effect of antibiotics (Ayaz et al., 2015). Fluoxetine shows a strong dose-dependent antimicrobial activity *in vitro* against *L. rhamnosus* and *E. coli* (Cussotto et al., 2018). An *in vivo* study on rats on fluoxetine found that this antidepressant completely inhibited the growth of *Succinivibrio* and *Prevotella* taxa (Cussotto et al., 2018). Although the role of its effect on antidepressant properties may be contrary, its side effects are still not clear. A recent study on mice treated with one of five different antidepressants (i.e., fluoxetine, escitalopram, venlafaxine, duloxetine, or desipramine) revealed that all the drugs except desipramine reduced richness (i.e., the variation of microbes in a single sample) of mice gut microbiota, while simultaneously increasing beta diversity (i.e., the variation of microbial communities between samples). This observation can cause concern because it is generally accepted that reduced microbiota richness is more common in conditions such as IBS and obesity. The main aim of this study, however, was to identify bacteria directly influencing the antidepressant mechanism of study drugs. Based on a series of experiments, the authors chose duloxetine and *Ruminococcus flavefaciens* and found that the supplementation of *R. flavefaciens* reduced or even abolished antidepressive and antianhedonic properties of duloxetine. Further investigation has shown that the mechanism of these bacterial actions may involve impairment of mitochondrial oxidative phosphorylation and neural plasticity in medial prefrontal cortex (mPFC). This can be explained by the gene expression changes in synaptic and mitochondrial genes, which are induced by *R. flavefaciens*. Therefore, the dysregulated mitochondrial function and decreased neuroplasticity in mPFC could contribute to *R. flavefaciens* attenuation of antidepressant effects on depressive-like behavior. Most importantly, the authors found reduced levels of serotonin and noradrenaline in mPFC as a result of *R. flavefaciens* supplementation (Lukić et al., 2019).

## Antibacterial Effect of Ketamine

The acute effect of ketamine includes inhibition of NMDARs and the activation of AMPARs, as well as molecular signaling of the mammalian target of rapamycin (mTOR), which results in the enhancement of hippocampal (BDNF) and increased synaptogenesis (Pałucha-Poniewiera, 2018). Apart from these mechanisms, ketamine also has antimicrobial properties, as observed in *in vitro* studies. In an earlier study by Gocmen et al., ketamine was used in high anesthetic doses, and it has shown prominent antibacterial effect against six different strains of bacteria, namely, *S. aureus, Staphylococcus epidermidis, Enterococcus faecalis, Streptococcus pyogenes, P. aeruginosa*, and *E. coli*. The authors pointed out that the doses used are not for human or anesthetic purposes, so this antibacterial effect is not possible to observe *in vivo* (Gocmen et al., 2008). In a similar study, the antimicrobial effect of ketamine against *S. aureus, E. coli*, and *P. aeruginosa* was confirmed when ketamine was used with propofol [i.e., a mixture called ketofol used for electroconvulsive therapy (ECT) anesthesia] and when ketamine was suggested as safe anesthesia for surgical approaches (Begec et al., 2013). In a more recent study on radish seeds treated with sub-anesthetic doses of ketamine, the authors observed the antibacterial effect on *S. epidermidis* and *Borrelia burgdorferi*. They concluded that the possible mechanism behind this effect could remain in l-glutamate signaling networks and NMDAR ion channels of bacteria (Torres et al., 2018).

## Anti-Inflammatory Effect of Ketamine

Ketamine has an anti-inflammatory effect in depression (Szałach et al., 2019). The study which included patients with TRD indicated that pro-inflammatory cytokines (mainly IL-6, G-CSF, and IL-1α) reduced 4 h after a single dose of intravenous (IV) ketamine (Kiraly et al., 2017). Another study has shown rapid decreases in levels of IL-6 and TNF-α, as well as a correlation between the decrease in TNF-α and a reduction in the Montgomery–Asberg Depression Rating Scale (MADRS) score (Chen et al., 2018). Kadiru et al. found that ketamine influences the kynurenine pathway by increasing the level of kynurenine and kynurenic acid and decreasing the level of quinolinic acid acting as a rapid anti-inflammatory agent in patients with bipolar depression (Kadriu et al., 2019). In another trial, treatment with 6 ketamine IV infusions in patients with MDD correlated with the elevation of kynurenine and tryptophan; moreover, the downregulation of inflammation was observed and the authors suggested that the anti-inflammatory effect of ketamine may contribute to its rapid antidepressant effect (Kadriu et al., 2019). Moreover, stress-related changes in the gut microbiome affect BDNF concentration and NMDAR activity (Baj et al., 2019), and ketamine may hypothetically reverse these changes. Walker et al. investigated the effect of ketamine in LPS-induced depression and found that ketamine abrogated LPS-induced depressive behaviors by antagonizing NMDA activation (Walker et al., 2013). One of recent studies investigated the relationship between hippocampal volume and inflammatory markers following six ketamine infusions in 44 patients with depression. The authors confirmed antidepressant effect of ketamine and increase in right hippocampal volume, but found no correlation with the change in concentrations of a group of inflammatory markers one day after ketamine treatment. Analyzing cytokine concentrations separately they confirmed significant changes in kynurenic acid, fractalkine, IFN-γ, TNF-α, IL-2, IL-4, IL-6, and IL-10 (Zhou et al., 2020). The abovementioned data suggest that ketamine may reach its antidepressant effect, at least partly, through inhibiting inflammatory reaction, which is one of the main elements of gut–brain interplay.

## Effect of Ketamine on Microbiome in Animal Models of Depression

The electronic databases, such as PubMed, MEDLINE, and EBSCO host, were searched for the following keywords and their combinations: ketamine, esketamine, arketamine, and gut microbiota; ketamine and gut microbiome; ketamine and gut bacteria; ketamine and GBA. We have also conducted cross-reference search based on the reports found.

We have identified four studies focusing on the effect of ketamine on gut microbiota in rodents subjected to CSDS—an animal model of depression. This model does not show treatment resistance, which is a significant fact, since most ketamine human studies involve treatment-resistant patients. Such a model has been used in a study on deep brain stimulation (Papp et al., 2018).

Nevertheless, we have found no studies on the ketamine effect on gut microbiota in treatment-resistant rodents. All studies with detailed descriptions are presented in Table 1. The first study investigated the role of microbiota in an antidepressant effect of two ketamine enantiomers, S- and R-ketamine (Yang et al., 2017b). The authors investigated both enantiomers since it has been reported that R-ketamine has greater potency and long-lasting antidepressant effects and has fewer adverse effects than S-ketamine (Yang et al., 2015). The results confirmed the antidepressant effect of both enantiomers as the increased immobility time in TST (tail suspension test) and FST (forced swimming test) was reduced after ketamine administration in susceptible mice. R-ketamine had stronger antidepressant and antianhedonic properties than S-ketamine. The observed increased levels of Actinobacteria in CSDS-susceptible mice is in line with the evidence that this phylum is increased in MDD (Jiang et al., 2015; Zheng et al., 2016); interestingly, neither of ketamine enantiomers modified this effect. The authors pointed out the possible role of Deltaproteobacteria and Desulfovibrionaceae in MDD as their presence in the gut correlates with an increased inflammatory response in humans. The number of genus *Butyricimonas* producing butyrate, which is known for anti-inflammatory potential, was restored mainly by R-ketamine after the CSDS, and this observation suggests the role of GBA in the antidepressant effect of R-ketamine (Yang et al., 2017b). In the second study, as a comparator for R-ketamine, the authors used lanicemine, which is also an NMDA antagonist, but it does not show antidepressant effect in humans (Sanacora et al., 2016). In line with this research, lanicemine did not cause any antidepressant effect contrary to R-ketamine. The authors observed alterations in gut microbiota after CSDS, which were attenuated by R-ketamine and to some extent also by lanicemine, although its effect was less potent. The most interesting effects of R-ketamine in this study were the restoration of Bacteroidales, previously reported as reduced in depression (Naseribafrouei et al., 2014), and Clostridiales (i.e., butyrate-producing bacteria) and reduction in genus *Clostridium*, previously reported as increased in MDD (Jiang et al., 2015; Qu et al., 2017). The third study investigated changes in gut microbiota composition after low-dose ketamine vs. placebo. The authors found a significant increase in *Lactobacillus*, which has been reported as a probiotic with antidepressant properties in animal studies (Getachew et al., 2018; Liu et al., 2019). They also observed the potent reduction of *Mucispirillum* and *Ruminococcus*, which are associated with inflammatory processes in the gut and IBS, and therefore, they suggested that the effect of ketamine may be mediated by these microorganisms (Getachew et al., 2018). High-level *Mucispirillum* can increase gut permeability and be responsible for high LPS concentration, which enhances inflammatory processes (Liu et al., 2019). Ruminococcaceae reduction was also observed in the earlier study (Qu et al., 2017). Another effect was the increase of *Turicibacter*, which is associated with an increase in butyric acid levels in the gut (Zhong et al., 2015). The most recent study investigated the role of gut microbiota in the antidepressive effect of ketamine in an inflammation model of depression. Ketamine had an antidepressant effect, when observed in behavioral tests, as it reduced immobility time, previously increased by LPS administration (Huang et al., 2019). It was found that an increase in the phylum Actinobacteria and class Coriobacteriia and order Clostridiales correlated with reduced immobility time. On the other hand, a decrease in family Prevotellaceae and the genus *Alloprevotella* correlated with the antidepressant effect of ketamine in LPS-treated mice (Huang et al., 2019). The authors also suggested that the phylum Actinobacteria and the class Coriobacteriia are potential biomarkers for the antidepressant effects of ketamine in an inflammation model.

**Table 1:** Studies on ketamine's effect on gut microbiota in rodent model of depression.

**References**	**Animal model and behavioral tests**	**Intervention**	**Microbiome after CSDS in susceptible mice**	**Effect of the intervention on microbiome**
Yang et al. (2017b)	Mice CSDS TST FST SPT	R-ketamine S-ketamine 10 mg/kg vs. saline	*Phylum* Tenericutes ↓ Actinobacteria ↑ *Class* Deltaproteobacteria ↑ Mollicutes ↓ *Family* Desulfovibrionaceae ↑ G*en*us Butyricimonas ↓	None None R and S-ketamine ↓ R-ketamine ↑ S-ketamine ↑ R,S-ketamine ↑ R-ketamine more potent
Qu et al. (2017)	Mice CSDS SIT TST FST SPT	R-ketamine 10 mg/kg vs. lanicemine 10 mg/kg vs. saline	*Order* Bacteroidales↓ Clostridiales↓ *Family* Ruminococcaceae↑ Mogibacteriaceae ↓ *Genus* Clostridium↑↑	R-ketamine ↑ R-ketamine ↑ R-ketamine ↓ lanicemine less potent R-ketamine ↓ lanicemine less potent
Getachew et al. (2018)	Rats No model of depression	Ketamine 2.5 mg/kg vs. saline	–	*Phylum* Deferibacteres ↓ Tenericutes↓ *Class* Deferrribacteres↓ Mollicutes↓ *Order* Turicibacterales↑ Desulfuromonadales↓ Deferribacterales↓ Theromonaerobacterales↓ Anaeroplasmatale↓ *Family* Tuberibacteraceae ↑ Clostridiaceae↑ Lactobacillaceae↑ Deferrribacteraceae↓ Ruminococcaceae↓ *Genus* Sarcina ↑ Turicibater↑ Lactobacillus↑ Mucispirillum↓ Ruminococcaceae↓
Huang et al. (2019)	Mice, LPS-induced inflammation model of depression FST Locomotion	Ketamine 10 mg/kg vs. saline	*Phylum* Actinobacteria **↓** Firmicutes **↓** *Class* Coriobacteriia **↓** Clostridia **↓** *Order* Clostridiales **↓** *Family* Prevotellaceae **↑** *Genus* Alloprevotella **↑** Butyricimonas **↑**	Ketamine **↑** Correlation with the effect of ketamine on FST in LPS mice: **Negative** *Phylum* Actinobacteria *Class* Coriobacteriia *Order* Clostridiales **Positive** *Family* Prevotellaceae *Genus* Alloprevotella

*SIT, social interaction test; TST, tail suspension test; FST, forced swimming test; SPT, Sucrose Preference Test; ↓, decreased number of bacteria; ↑, increased number of bacteria*.

Ketamine seems to restore bacteria producing the anti-inflammatory substance, butyrate, and reduce the number of bacteria associated with inflammatory processes in the gut. It increases the abundance of bacteria, previously reported as reduced in depression, and reduces the amount of bacteria, previously reported as increased in MDD. It also increases the abundance of probiotic bacteria known to produce an antidepressant effect.

These results are preliminary and should be treated with caution, although further investigation of this field may elucidate the mechanism of the antidepressant effect of ketamine and may facilitate further research and lead to the discovery of new treatment strategies in depressive disorders.

## Conclusion

The gut microbiome and the brain are involved in constant communication. This relationship undergoes specific changes in MDD. Previous studies validated the microbiome as a target for therapeutic intervention in this disorder. Ketamine is a novel rapid-acting antidepressant with antisuicidal properties. The mechanisms behind the therapeutic effect of ketamine are still not fully understood, although its anti-inflammatory properties potentially affect GBA in depression. There is a need for further studies on the effect of ketamine and its enantiomers on individual bacterial species. It is crucial to identify more species engaged in the inflammatory pathway of GBA to study their interactions with mediators of depression and to investigate how ketamine can affect them. There is also a need to reconsider animal models of depression used in these studies to investigate treatment resistance. Apart from animal studies, it will be necessary to conduct clinical studies on patients with MDD treated with ketamine with a detailed examination of microbiota alterations before and after the treatment and correlation with treatment outcome, no such studies have been published so far. Considering the impact of depression on human health, there is an unquestionable need for a better understanding of the role of the GBA in treating depressive symptoms. This understanding will hopefully lead to discovering novel antidepressants acting on microbiota and bring more effective and individualized treatment strategies for depressed patients.

## Author Contributions

AW: conceptualization, research, writing—original draft preparation, and editing. ŁS: writing, research, and drawing figures. WC: conceptualization, review and editing, and funding acquisition. All authors have read and agreed to the published version of the manuscript.

## Conflict of Interest

AW has received research support from Angelini, Biogen, Eli Lilly and Company, Janssen- Cilag, Lundbeck, Polpharma, Sanofi and Valeant. ŁS has received support from Angelini and +Pharma. WC has received research support from Actavis, Alkermes, Allergan, Angelini, Auspex, Biogen, Bristol-Myers Squibb, Cephalon, Eli Lilly, Ferrier, Forest Laboratories, Gedeon Richter, GW Pharmaceuticals, Janssen, KCR, Lundbeck, Orion, Otsuka, Sanofi, and Ser-vier; he has served on speakers bureaus for Adamed, Angelini, AstraZeneca, Bristol-Myers Squibb, Celon, GlaxoSmithKline, Janssen, Krka, Lekam, Lundbeck, Novartis, Orion, Pfizer, Polfa Tarchomin, Sanofi, Servier, and Zentiva; and he has served as a consultant for GW Pharmaceu-ticals, Janssen, KCR, Quintiles, and Roche.
